# Identification of a novel tumor angiogenesis inhibitor targeting Shh/Gli1 signaling pathway in Non-small cell lung cancer

**DOI:** 10.1038/s41419-020-2425-0

**Published:** 2020-04-14

**Authors:** Xueping Lei, Yihang Zhong, Lijuan Huang, Songpei Li, Jijun Fu, Lingmin Zhang, Yu Zhang, Qiudi Deng, Xiyong Yu

**Affiliations:** 1 Key Laboratory of Molecular Target & Clinical Pharmacology and the State Key Laboratory of Respiratory Disease, School of Pharmaceutical Sciences & the Fifth Affiliated Hospital, Guangzhou Medical University, Guangzhou, 511436 Guangdong China; 2 GMU-GIBH Joint School of Life Sciences & the Third Affiliated Hospital, Guangzhou Medical University, Guangzhou, 511436 China

**Keywords:** Tumour angiogenesis, Pharmacodynamics

## Abstract

Although angiogenesis inhibitors targeting VEGF/VEGFR2 have been applied for tumor therapy, the outcomes are still unsatisfactory. Thus, it is urgent to develop novel angiogenesis inhibitor for cancer therapy from new perspectives. Identification of novel angiogenesis inhibitor from natural products is believed to be one of most promising strategy. In this study, we showed that pristimerin, an active agent isolated from traditional Chinese herbal medicine *Celastrus aculeatus* Merr, was a novel tumor angiogenesis inhibitor that targeting sonic hedgehog (Shh)/glioma associated oncogene 1 (Gli1) signaling pathway in non-small cell lung cancer (NSCLC). We showed that pristimerin affected both the early- and late-stage of angiogenesis, suggesting by that pristimerin inhibited Shh-induced endothelial cells proliferation, migration, invasion as well as pericytes recruitment to the endothelial tubes, which is critical for the new blood vessel maturation. It also suppressed tube formation, vessel sprouts formation and neovascularization in chicken embryo chorioallantoic membrane (CAM). Moreover, it significantly decreased microvessel density (MVD) and pericyte coverage in NCI-H1299 xenografts, resulting in tumor growth inhibition. Further research revealed that pristimerin suppressed tumor angiogenesis by inhibiting the nucleus distribution of Gli1, leading to inactivation of Shh/Gli1 and its downstream signaling pathway. Taken together, our study showed that pristimerin was a promising novel anti-angiogenic agent for the NSCLC therapy and targeting Shh/Gli1 signaling pathway was an effective approach to suppress tumor angiogenesis.

## Introduction

Angiogenesis is a process of new blood vessel sprouting from the pre-existing endothelium. It is critical for the growth and development of malignant cancer^1,2^. New blood vessels furnish tumor with oxygen and nutrients, infiltrate the tumor cells, and provide routes for their metastasis. Therefore, angiogenesis blockade has been identified as an effective therapeutic strategy for multiple types of cancers, including breast cancer, colorectal cancer and non-small cell lung cancer (NSCLC)^3–5^.

Angiogenesis is a highly complex process, including destabilization of the integrated blood vessel, proliferation, migration, invasion, and tubulogenesis of endothelial cells as well as pericytes attachment to newly formed blood vessel. And it is regulated by multiple pro-angiogenic and anti-angiogenic factors, such as vascular endothelial growth factor (VEGF), fibroblast growth factor (FGF), growth arrest- specific protein 6 (Gas6), platelet-derived growth factor (PDGF)^6–8^. Of all the known angiogenic molecules, VEGF and its receptor VEGFR2 have been recognized as one of the most important targets for anti-angiogenic therapy of cancer. Many monoclonal antibodies and inhibitors targeting VEGF/VEGFR2 have been developed and applied for NSCLC therapy as a single agent or combined with other chemotherapy drugs, including bevacizumab, ramucirumab, sunitinib and sorafenib. However, the result is still unsatisfactory, the 5-year survival rate of patients with angiogenesis inhibitor targeting VEGF/VEGFR2 are still poor^9–11^. All these suggest that it is urgent to develop novel angiogenesis inhibitor with low toxicity for NSCLC therapy from new perspective.

Recently, sonic hedgehog (Shh) has been recognized as a novel pro-angiogenic factor. It is overexpressed in multiple malignant tumors, including breast cancer, glioma and pancreatic adenocarcinoma^12–14^. After binding to its receptor Patched (PTCH), Shh induces release of smoothened (SMO), which mediates downstream activation of the transcription factor glioma associated oncogene 1 (Gli1), thereby regulating various target genes that involve in cell growth, differentiation, drug resistance and angiogenesis^15–17^. Shh-mediated activation of Gli1 contributes to the proliferation, migration, invasion and tube formation of endothelial cells, as well as the maturation of newly formed blood vessel^18,19^. Activation of Shh/Gli1 signaling pathway has been observed during angiogenesis process in many pathologic conditions, including myocardial ischemia, coronary vascular development and cancer^20^. Administration of Shh and activation of Shh/Gli1 signaling pathway stimulate angiogenesis in myocardial and stroke^21^. VEGF/VEGFR2, Ang/Tie2 and PDGF/PDGFR2 signaling pathway have been certified to be involved in Shh-mediated angiogenesis^22–24^. In addition, several Shh/Gli 1 signaling pathway inhibitors have been demonstrated to suppress tumor growth and angiogenesis^25,26^.

Pristimerin, an active agent that isolated from traditional Chinese herbal medicine *Celastrus aculeatus* Merr, poses multiple pharmacological activities, such as anti-inflammatory, antiperoxidative and antimicrobial^27–29^. Pristimerin also exhibits perfect anti-tumor effect in multiple types of cancers (colorectal cancer, breast cancer and prostate cancer) via inducing cell cycle arrest, apoptosis, necrosis and incomplete autophagy. Pristimerin also inhibits VEGF-induced endothelia cellular motilities and angiogenesis in adjuvant-induced arthritic rats by blocking VEGF/VEGFR2 signaling pathway^27,30^. However, its effect on tumor angiogenesis and Shh/Gli1 signaling pathway are still unclear. In the present study, we showed that pristimerin inhibited Shh-induced endothelial cellular motilities (including proliferation, migration, invasion and tube formation) and pericytes recruitment to newly formed endothelial cells tubes, indicating that pristimerin affected both the early- and late-stage of angiogenesis. Pristimerin also significantly decreased microvessel density (MVD) and pericytes coverage in NCI-H1299 xenograft model. Further research showed that pristimerin restrained the activation of Shh/Gli1 and its downstream signaling pathway. Our study indicates that pristimerin might serve as a promising anti-angiogenic agent for NSCLC therapy and Shh/Gli1 signaling pathway could be a potent anti-angiogenic target for NSCLC therapy.

## Materials and methods

### Material

Pristimerin with a purity of 98% was obtained from SelleckChem (Houston, Texas, USA) and then dissolved in DMSO to produce a 20 mM stock solution. Endothelial cell medium (ECM) and pericyte medium (PM) were obtained from ScienCell Research Laboratories (San Diego, CA, USA). Cell Counting Kit-8 (CCK-8) was from Beyotime (Shanghai, China). Pentobarbital sodium was obtained from Merck (Darmstadt, Germany). Matrigel was purchased from BD Biosciences (Franklin Lakes, USA). Recombinant human CellExp™ Shh was purchased from BioVision (Palo Alto, CA, USA). Antibodies against α-SMA (19245S), Ki67 (9449T), Akt (4685S), p-Akt^Thr308^ (13038S), ERK (4695S), p-ERKT^hr202/Tyr204^ (4370T), PDGFR-β (3174T), p-PDGFR-β^Tyr857^ (3170T), β-actin (4970T), Alexa Fluor 594 Donkey anti-Goat IgG (889S), Alexa Fluor 488 Donkey anti-Rabbit IgG (4416S) and HRP-conjugated anti-rabbit IgG antibody (4412S) were purchased from Cell Signaling Technology (Danvers, MA, USA). Antibodies against Gli1 (AF3455), CD31 (AF3628), and lamin B (MAB8525) antibody was obtained from R&D Systems (Minneapolis, MN, USA). PKH 26, PKH 67 and other regents were purchased from Sigma-Aldrich (St. Louis, MO, USA).

### Cell lines and culture

Human umbilical vein endothelial cells (HUVEC), Human microvascular endothelial cell line (HMEC-1), and Human brain vascular pericytes (HBVPs) were obtained from ScienCell Research Laboratories. HUVECs and HMEC-1 cells were cultured in ECM, and HBVPs were cultured in PM. The human non-small lung cancer cell line NCI-H1299 cells (it is isolated from a NSCLC patient with lymph node metastasis who have received prior radiation therapy and has a homozygous partial deletion of the p53 protein) were purchased from American Type Culture Collection (ATCC, Manassas, Virginia) and cultured with DMEM supplemented with 10% FBS and 1% penicillin/streptomycin. All these cells were maintained in humidified atmosphere containing 5% CO_2_ at 37 °C. We showed that all these cells have no cross contamination of other human cell lines using the STR Multi-Amplification Kit (Microreader 21 ID System).

### Animals

Male BABL/c (nu/nu) mice were purchased from Hua Fukang Experimental Animal Center (Beijing, China). Adult female Sprague Dawley rats were obtained from Guangdong Medical Experimental Animal Center (Guangzhou, China). All animals were maintained in aseptic conditions with constant humidity and temperature. All animals’ experiments were approved by Laboratory Animal Ethics Committee of Guangzhou medical university according with accordance with the ARRIVE (Animal Research: Reporting In Vivo Experiments) guidelines.

### Cell proliferation assay

The cell ability was evaluated with CCK-8 assay. HUVECs and HMEC-1 cells (0.5 ×10^5^) were seeded in 96-well plate. After 24 h, the cells were treated with Shh and different concentrations of pristimerin for another 24 h. And then 10 μL of CCK-8 reagent were added to the cells and incubated for 4 h at 37 °C, the absorbance was determined by a SynergyMx Multi-Mode Microplate Reader (Biotek, Winooski, VT) at 450 nm. The experiment was conducted three times independently.

### Wound-healing assay

HUVECs and HMEC-1 cells (5 × 10^5^) were seeded in six-well plate and cultured until the cells grew to confluent. After starving with serum-free ECM for 6 h, the cells were scratched with pipette tips. The cells were treated with Shh and various concentrations of pristimerin in ECM containing 1% FBS for 8 h, and images of the same fields were taken at 0 and 8 h with an EVOS XL Core (Thermo Fisher Scientific). The migratory cells were quantified with Image-Pro Plus 6.0 software (Media Cybernetics, Rockville, MD, USA). The experiment was conducted thrice independently.

### Transwell migration and invasion assay

The effect of pristimenrin on Shh-mediated HUVECs and HMEC-1 cells migration and invasion was evaluated by transwell migration and invasion assay using transwell chambers (8 μm pore size, Corning), respectively. In the transwell migration assay, HUVECs and HMEC-1 cells suspended with serum-free ECM were seeded in the upper chamber of the plates at the density of 2 × 10^4^ per well, and the bottom chamber was filled with 0.6 mL fresh ECM. The cells were cultured for 24 h at 37 °C. After removing the non-migratory cells on the surface of the upper membrane with cotton tip, the cells migrated to the lower membrane surface were fixed by 4% paraformaldehyde and stained using 0.1% crystal violet. The images were taken using an EVOS XL Core with three randomly selected fields in each well, and migratory cells were analyzed using Image-Pro-Plus 6.0.

For the transwell invasion assay, all the steps were carried out similarly to that in the transwell migration assay except that the upper chambers were pre-coated with Matrigel. The experiment was repeated three times independently.

### Network formation assay

HUVECs and HMEC-1 cells were seeded in Matrigel-coated 96-well plate at the density of 2 × 10^4^ per well in the presence or absence of Shh and pristimenrin. And the cells were incubated at 37 °C with 5%CO_2_ for 6 h. The networks were observed and captured using an EVOS XL Core, and the tube numbers were measured and analyzed with Image-Pro-Plus 6.0. The experiment was replicated thrice.

### Adhesion assay

The HBVP cells pretreated with or without Shh and pristimenrin were seeded in the 24-well plates. The cells were allowed to attach for 2 h in fresh culture medium, and then were fixed with 4% paraformaldehyde. After that, the cells were stained using 0.1% crystal violet. The pictures were captured with an Olympus BX53 upright microscope and the adhesion cells were quantified by Image-Pro Plus 6.0. Three experiments were performed independently.

### Co-culture tubule-like structure of endothelial cells and pericytes

The HUVECs and HBVPs were stained with PKH26 (red) and PKH67 (green) fluorescent dyes respectively to track relative endothelial cells and pericytes. HUVECs (3 × 10^4^ per well) were seeded in Matrigel pre-coated 96-well plate and cultured for 2 h to form a capillary network. HBVPs (1 × 10^4^ per well) pretreated with Shh and pristimentrin were added to the endothelia capillary network and cultured for another 10 h. The tube structures were observed and captured with EVOS XL Core at 0, 1, and 10 h since the addition of HBVPs. The experiment was repeated three times independently.

### Aortic ring assay

The aortic ring assay was conducted to determine the anti-angiogenic effect of pristimentrin in according to previous study^31^. Briefly, the thoracic aorta was isolated from Sprague Dawley rats and rinsed in PBS. The thoracic aorta was cut into 1- to 1.5-mm-long rings after excising fibroadipose tissue, and the rings were seeded in Matrigel pre-coated 96-well plate and layered with another 80 μL Matrigel. After incubating for 2 h, the aortic rings were treated with fresh ECM containing Shh and various concentrations of pristimentrin for 6 days. The sprouted microvessels were observed and photographed using EVOS XL Core. The MVD was quantified using ImageJ software. The experiment was repeated three times independently.

### Chorioallantoic membrane assay

We conducted chorioallantoic membrane (CAM) assay with fertilized chicken eggs that have been incubated at 37 °C in a humidified incubator containing 60–65% humidity for 5 days according to previous described^32^. A small window ~1 cm^2^ was cut at the eggshell on the gas chamber side to develop an artificial gas chamber. The shell and shell membrane were detached to expose the CAM. And then the CAMs were randomly divided into vehicle group, pristimentrin 0.2 mg/kg group and pristimentrin 0.4 mg/kg group with eight eggs per group. After sealing the window with transparent tape, the eggs were incubated for another 48 h. The blood vessels in CAM were observed and captured using an Olympus SZX18 dissecting microscope, and the microvessels were quantified by Image-Pro Plus 6.0.

### Immunofluorescence staining

HUVECs and HMEC-1 cells were treated with or without Shh and pristimentrin and then fixed with 4% paraformaldehyde. The fixed cells were permeabilized with 0.1% Triton X-100 and blocked with 5% BSA. After that, the cells were labeled with the primary antibodies at 4 °C overnight, following by incubating Alexa Fluor 488 Donkey anti-Rabbit IgG at room temperature for 1 h. And then the cells were counterstained with DAPI to recognize the nucleus. For the immunofluorescence assay of the tumor tissues, the tumor sections were blocked with 5% BSA and incubated with CD31 antibody and PDGFR-β antibody overnight at 4 °C. After washing with PBS, the sections were stained with Alexa Fluor 488 Donkey anti-Rabbit IgG and Alexa Fluor 594 Donkey anti-Goat IgG for 1 h at room temperature, following with DAPI staining to recognize the nucleus. The images were obtained using a laser scanning confocal microscope (LSM 800, ZEISS) with a ×63 objective.

### Western blotting assay

HUVECs, HMEC-1 cells and HBVPs that treated with or without Shh and pristimentirn were lysed using RIPA buffer to obtain whole-cell extracts. The cytoplasmic and nucleus proteins were isolated with a NE-PERTM Nucleus and Cytoplasmic Extraction Kit (ThermoFisher Scientific) according to the manufacturer’s protocol. The total protein concentration was determined with BCA Protein Assay kit (Beyotime Biotechnology, China). And then the proteins were separated by 8–12% SDS-polyacrylamide gels and transferred onto the polyvinylidene difluoride membranes (Millipore, Bedford, MA, USA). After that, the membranes were incubated with antibodies and detected using enhanced chemiluminescence detection system. Quantitative data was measured with ImageJ software (NIH, NY). The experiment was repeated thrice independently.

### In vivo mouse xenograft assay

Four-to-six-week male nude mice (BALB/cA-nu) were subcutaneously inoculated with NCI-H1299 cells (1 × 10^6^) suspended in 100 µl Matrigel into the right flank. When the tumor grew to ~100 mm^3^, tumor-bearing mice were randomly divided into three groups with five mice per group. The mice in each group were intraperitoneal injected with vehicle, pristimentrin 0.2 mg/kg and pristimentrin 0.4 mg/kg, respectively. The tumor volume was monitored and calculated on the base of the formula (0.5 × width^2^ × length) using two perpendicular measurements with a caliper. At the end of the experiment, the mice were anesthetized using pentobarbital sodium. The tumors were excised, weighted and photographed. And then, the tumors were fixed with 4% paraformaldehyde for further histological and immunohistochemical assay.

### Histology and Immunohistochemistry assay

After being fixed in 4% paraformaldehyde for 24 h, the tumors were embedded in paraffin and then cut into 5-μm-thick sections. The sections were incubated with hematoxylin-eosin (H&E) according to the standard procedures. For immunohistochemistry assay, the sections were incubated with primary antibodies (Ki67, CD31, Gli1 and VEGFR2) and then detected by HRP-conjugated secondary antibody using a DAB kit. The images were visualized and taken with a Carl Zeiss fluorescence microscope. And the semiquantitative image analysis was performed with Image Pro Plus software.

### Statistical analysis

The data was analyzed with GraphPad Prism 5.0 (GraphPad Software, Inc., San Diego, CA) and presented as the mean ± SEM. Significant differences were performed using one-way ANOVA followed by a Tukey’s test and *p* < 0.05 was considered to be statistically significant.

## Results

### Pristimerin inhibits Shh-induced HUVECs and HMEC-1 cells proliferation, migration, and invasion

**Fig. 1:**
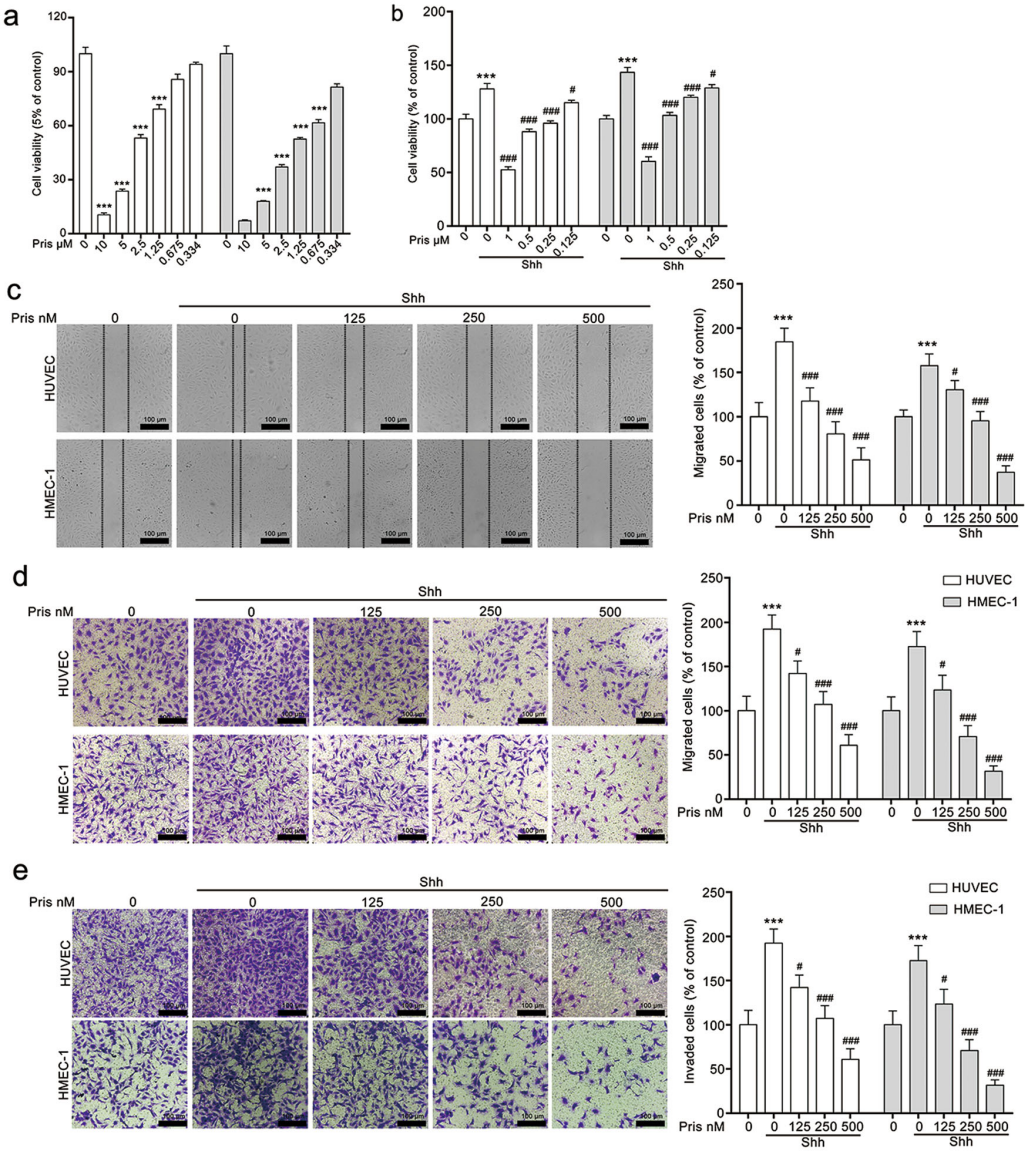
Pristimerin inhibits Shh-induced proliferation, migration, adhesion and invasion of HUVECs and HMEC-1 cells. **a** Pristimerin suppresses cell proliferation of HUVECs and HMEC-1 cells in a dose-dependent manner. The cells were treated with various concentration of pristimerin for 48 h, and the cell viability was detected using CCK-8 assay. **b** Pristimerin treatment reduced Shh-induced cell viabilities of HUVECs and HMEC-1 cells. After starving with serum-free medium, the cells were treated with or without Shh (100 ng/mL) and various concentrations of pristimerin (125, 250, and 500 nM) for 48 h, and the cell viability was detected using CCK-8 assay. **c** Pristimerin restrains Shh-induced horizontal migration of HUVECs and HMEC-1 cells. After being starved with non-serum ECM, the wound was generated with pipette tips in the monolayers of HUVECs and HMEC-1. And the cells were exposed to Shh and different concentrations of pristimerin for 10 h. And the images were photographed at 0 and 10 h. Representative images were shown and the quantification of migrated cells was presented at the right panel. Scale bar: 100 μm. **d**, **e** Pristimerin restrains Shh-induced migration and invasion of endothelial cells. The cells were seeded in the upper chamber of transwell that have pre-coated with or without Matrigel, and then the cells were exposed to Shh (100 ng/mL) and various concentrations of pristimerin for 24 h. After removing the cells in the inner of upper chamber, the migrated cells and invaded cells were photographed. And the representative images of migrated cells and invaded cells were presented in **d**, **e**, respectively. Scale bar: 100 μm. The data are presented as mean ± SEM, *n* = 3. ^***^P < 0.001 compared with the control group; ^#^*P* < 0.05 and ^###^*P* < 0.001 compared with the Shh-treated group.

We first assessed the anti-angiogenic effect of pristimerin on endothelial cells with proliferation, migration and invasion assay. The cell viability assay was conducted to provide a rational concentration for further in vitro experiments. The result showed that pristimerin lower than 500 nM was non-toxic to HUVECs and HMEC-1 cells (Fig. 1a). And we selected 500, 250, and 125 nM for further in vitro and ex vivo anti-angiogenic experiments. Next, we examined the inhibitory effect of pristimerin on Shh-induced endothelia cell proliferation. The results showed that Shh (100 ng/mL) increased HUVECs and HMEC-1 cells viability, whereas pristimerin treatment decreased Shh-induced HUVECs and HMEC-1 cells viability in a dose-dependent manner (Fig. 1b). We also ascertained the effect of pristimerin on endothelial cells with wound-healing assay, tranwell migration and invasion assay. We found that Shh effectively increased the migration (horizontal and vertical) of HUVECs and HMEC-1 cells. Whereas, pristimerin treatment dramatically inhibited Shh-mediated migration (Fig. 1c, d). Similar results were observed in transwell invasion assay. Shh stimulation significantly promoted the invasion ability of HUVECs and HMEC-1 cells, but pristimerin treatment suppressed Shh-induced invasion (Fig. 1e). Taken together, these data suggests that pristimerin poses potent anti-angiogenic effect.

### Pristimerin suppresses Shh-induced angiogenesis ex vivo and in vivo

**Fig. 2:**
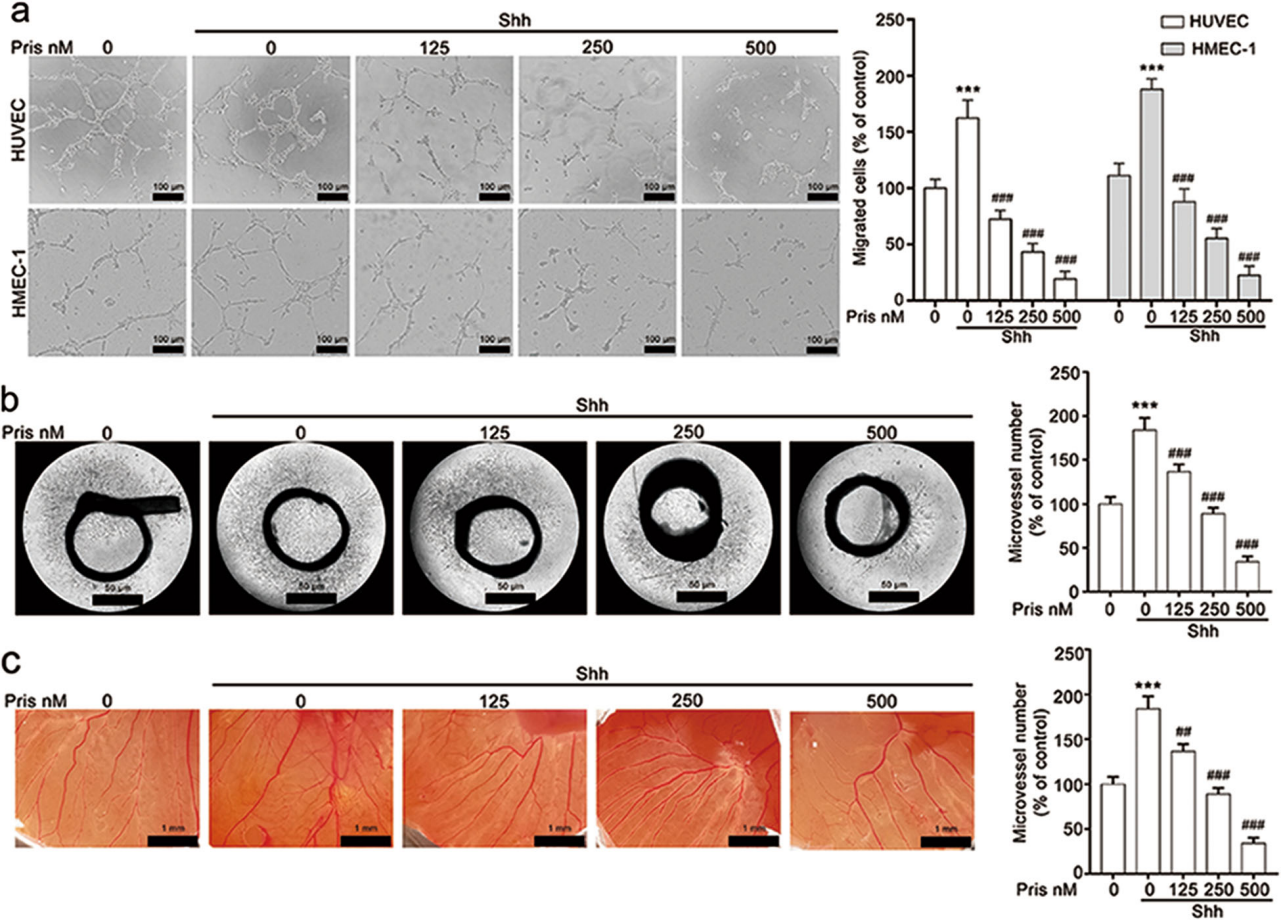
Pristimerin inhibits Shh-induced angiogenesis ex vivo and in vivo. **a** Pristimerin suppresses Shh-induced tube formation capabilities of HUVECs and HMEC-1 cells. The cells were seeded at Matrigel-coated 96-well plate and treated with or without Shh (100 ng/mL) and pristimerin (125, 250, and 500 nM) for 6 h. The endothelial tubes were observed and photographed at 6 h. And the quantitative of tubes were presented at the right panel. Scale bar: 100 μm, *n* = 3. **b** Pristimerin inhibits vessel sprouting in the rat aorta rings. Aorta rings that separated from Sprague Dawley rats were placed in the Matrigel-covered 96-well plate, and stimulated with Shh in the presence or absence of pristimerin. Representative images and quantitative data were shown. Scale bar: 50 μm, *n* = 3. **c** Pristimerin suppresses neovascularization in CAM model. Fertilized eggs were exposed to various concentrations of pristimerin in the presence or absence of Shh. And the neovessels were observed and photographed at 48 h. Scale bar: 1 mm, *n* = 8. The data are presented as mean ± SEM. ^***^P < 0.001 compared with the control group; ^##^*P* < 0.01, and ^###^*P* < 0.001 compared with the Shh-treated group.

To further validate the anti-angiogenic effect of pristimerin, we performed network formation assay and aortic ring assay. In the network formation assay, we found that Shh effectively promoted tube formation of HUVECs and HMEC-1 cells, evidenced by the number of tube-like structures increased and the tube-like structures was more stabilized in Shh-treated group. Whereas, the number of tube-like structures decreased and the tube-like structures was destabilized in pristimerin treated group (Fig. 2a). Similarly, aortic ring assay also showed that Shh stimulated new vessel sprouting obviously, indicated by the formation of a complex network of microvessels around the aortic rings in Shh group. However, pristimerin treatment significantly decreased vessel sprouting in a dose-dependent manner (Fig. 2b). Additionally, pristimerin treatment led to tortuous microvessels and fewer angiogenic vessels contacting the disk in the CAM when compared with that in Shh group (Fig. 2c). Based on these data, pristimerin suppresses Shh-induced angiogenesis ex vivo and in vivo.

### Pristimerin suppresses Shh/Gli1 signaling pathway

**Fig. 3:**
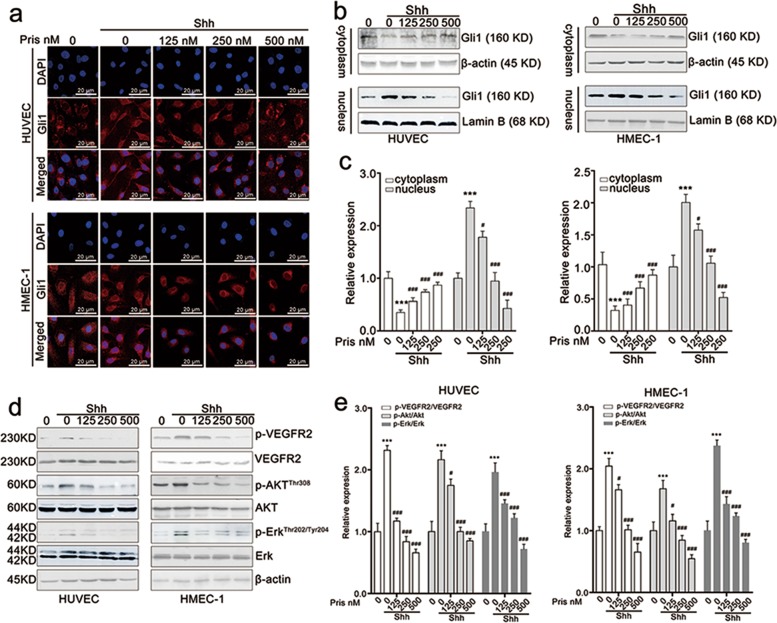
Pristimerin suppresses endothelial cellular motilities required for angiogenesis through inactivating Shh/Gli1 signaling pathway. **a** Pristimerin suppressed nucleus translocation of Gli1 in endothelial cells. After incubating with Shh (100 ng/mL) and pristimerin (125, 250, and 500 nM) for 24 h, the HUVECs and HMEC-1 cells were stained with Gli1 antibody and Alexa Fluor 488 Donkey anti-Rabbit IgG. DAPI was used to label the nucleus. The cells were observed and photographed with a laser scanning confocal microscope. Scale bar: 20 μm. **b**, **c** Pristimerin inhibits Gli1 distribution in endothelial cells. The HUVECs and HMEC-1 cells were incubated with Shh and pristimerin for 24 h, and then lysed with a NE-PERTM Nucleus and Cytoplasmic Extraction Kit. After that, proteins were subjected to western blotting assay. β-actin and Lamin B were set as loading control. The quantitative data were presented as ratios of Gli1/β-actin and Gli1/Lamin B. The representative blot and quantitative data were shown in B&C, respectively. **d**, **e** Pristimerin inactivated Shh/Gli1 and its downstream signaling pathway. The cells were treated with Shh and pristimerin for 24 h and lysed using RIPA buffer. Total proteins were subjected to western blotting assay. The representative blot and quantitative data were shown in **d**, **e**, respectively. The data are presented as mean ± SEM, *n* = 3. ^***^P < 0.001 compared with the control group; ^#^*P* < 0.05 and ^###^*P* < 0.001 compared with the SHH-treated group.

The above results showed that pristimerin exhibited perfect inhibitory effect on Shh-induced angiogenesis. And then we further investigated the effect of pristimerin on Shh/Gli1 signaling pathway. The immunofluorescence assay results showed that Shh stimulation promoted the translocation of Gli1 from cytoplasm to nucleus, whereas pristimerin treatment significantly reduced Gli1 distribution in nucleus (Fig. 3a). Western blotting assay also demonstrated that Shh stimulation increased the distribution of Gli1 in nucleus, and pristimerin treatment obviously decreased Shh-induced Gli1 distribution in nucleus (Fig. 3b, c). We also found that pristimerin treatment significantly blocked the Shh-mediated phosphorylation of VEGFR2, Akt and Erk in endothelial cells (Fig. 3d, e). All these results suggest that pristimerin suppresses endothelial cells mobility for angiogenesis through inactivating Shh/Gli1 and its downstream signaling pathway.

### Pristimerin attenuates the adhesion and recruitment of pericytes to the endothelial tubes

**Fig. 4:**
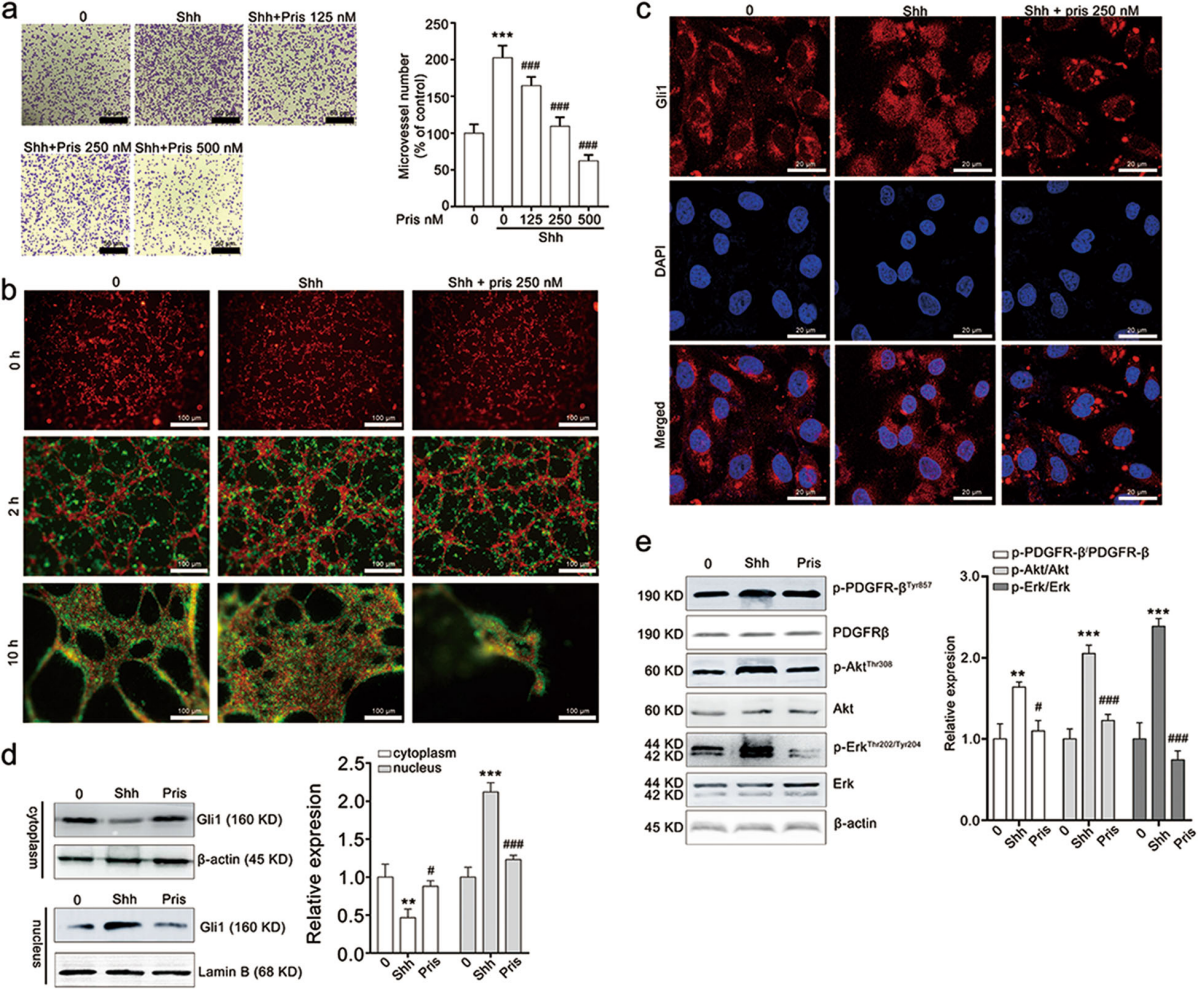
Pristimerin treatment decreases Shh-induced pericyte motilities of angiogenesis. **a** Pristimerin suppresses Shh-mediated adhesion ability of endothelial cells. The cells that pre-treated with Shh (100 ng/mL) and pristimerin (125, 250, and 500 nM) were seeded at 24-well plate. After 1-h culture, the un-attached cells were removed and the attached cells were stained with 0.1% crystal violet. **b** Pristimerin restrains Shh-induced pericyte migration to newly formed networks in a three-dimensional co-culture system of HUVEC and HBVP. HUVECs (red) were seeded on Matrigel-coated 96-well and cultured for 2 h to allow the formation of tube network. And HBVPs (green) pre-treated with or without pristimerin were added to the endothelial tubes. The recruitment of HBVPs to HUVEC tubes were observed and photographed at 0, 2, and 12 h. Scale bar: 20 μm. **c** Pristimerin suppresses the nucleus translocation of Gli1in HBVPs. HBVPs were stimulated with Shh (100 ng/mL) and pristimerin for 24 h. After staining with 4% paraformaldehyde, the HBVPs were labeled with Gli1 antibody and Alexa Fluor 488 Donkey anti-Rabbit IgG. DAPI was used to recognize the nucleus, and the images were taken with a laser scanning confocal microscope. **d** Pristimerin inhibits the distribution of Gli1 in the nucleus. HBVPs were collected and lysed with a NE-PERTM Nucleus and Cytoplasmic Extraction Kit following the manufacturer’s protocol, and then applied for western blotting assay. β-actin and Lamin B were set as loading control. **e** Pristimerin blocks the downstream of Shh/Gli1 signaling pathway. After being treated with Shh and pristimerin for 24 h, HBVP cells were lysed using RIPA buffer and subjected to western blotting assay. β-actin was set as loading control. The data are presented as mean ± SEM, *n* = 3. ^***^P < 0.001 compared with the control group; ^#^*P* < 0.05 and ^###^*P* < 0.001 compared with the Shh-treated group.

Considering that pericytes recruitment to the newly formed endothelial cells networks is critical for new blood vessel maturation during the angiogenesis late-stage^33^, we performed adhesion assay and 3D coculture assay of HUVECs and HBVPs to ascertain the effect of pristimerin on pericyte recruitment. The result showed that Shh stimulated a dramatic increase of HBVPs adhesion, and pristimerin treatment attenuated Shh-induced HBVPs adhesion (Fig. 4a). Similar results were observed in the 3D coculture assay, Shh promoted HBVPs recruitment to the newly formed networks, as evidenced by almost all HBVPs migrated to the endothelial tubes at 2 h and the networks were complex and solid in Shh group after 10 h incubation. By contrast, fewer HBVPs cells migrated to endothelial tubes within 2 h and the tubes regressed at 10 h in pristimerin treated group (Fig. 4b). All these showed that pristimerin restrained Shh-induced HBVPs’ recruitment to endothelial tubes. Further studies showed that pristimerin also reduced Shh-mediated Gli1 distribution in nucleus, indicated by that Shh stimulation promoted the nucleus translocation of Gli1 and pristimerin treatment suppressed this process (Fig. 4c, d).We also found that pristimerin inhibited Shh-induced phosphorylation of PDGFR, Akt and Erk in HBVPs (Fig. 4e). These data suggest that pristimerin could also inhibit the maturation of new blood vessel, which is different from previous angiogenesis inhibitors.

### Pristimerin suppresses tumor growth and tumor angiogenesis in NCI-H1299 xenografts

**Fig. 5:**
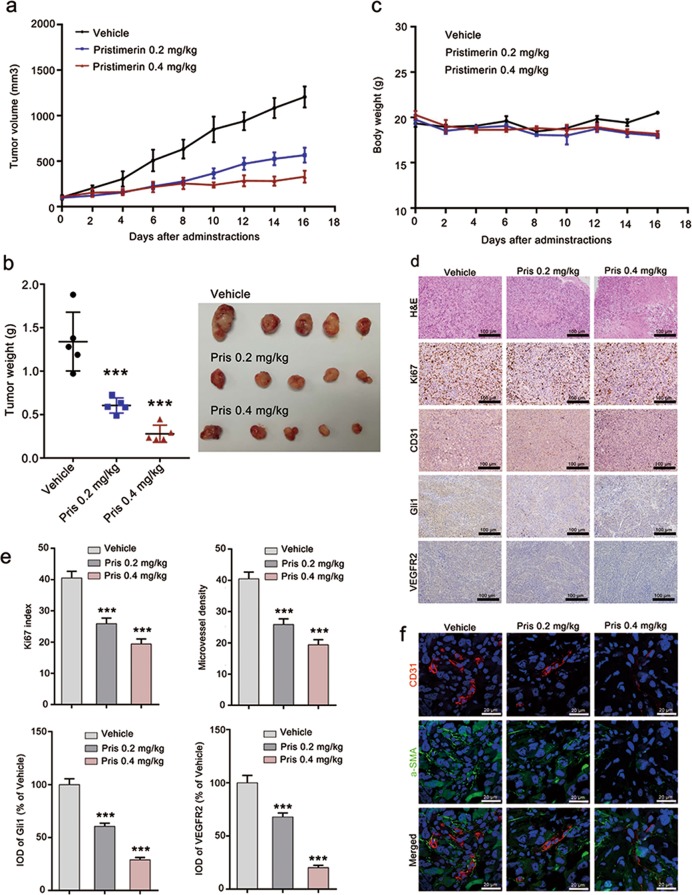
Pristimerin restrains tumor growth and angiogenesis in NCI-H1299 xenografts. **a**, **b** Pristimerin inhibits tumor growth in NCI-H1299 xenograft model. NCI-H1299 cells (1 × 10^7^ cells per mouse) were injected subcutaneously into 5–6-week-old male BABL/c (nu/nu) mice. When the tumor grew to ~200 mm^3^, the tumor-bearing mice were divided into vehicle group, pristimerin 0.2 mg/kg group and pristimerin 0.4 mg/kg group with five mice per group. And the mice were intraperitoneally injected (ip) with saline, 0.2 mg/kg of pristimerin and 0.4 mg/kg of pristimerin every two days for totally 16 days. And the tumor volumes were measured at the same time. At the end of the experiment, the mice were sacrificed and the tumor were removed, weighted and photographed. The tumor volume curve and tumor weight were presented at **a**, **b**, respectively. **c** Pristimerin has no significant effect on the body weight of the mice. **d**, **e** Pristimerin suppresses tumor angiogenesis through inhibiting Shh/Gli1 signaling pathway. Representative images of the H&E staining, immunohistochemistry and immunofluorescence of tumors were shown in **d**. Quantification of H&E staining, immunohistochemistry and immunofluorescence were analyzed using Image-Pro Plus 6.0 software. The data are presented as mean ± SEM and presented in **e**, *n* = 5, Scale bar: 100 μm. ^***^P < 0.001 compared with the Vehicle group. **f** Pristimerin decreases pericyte coverage in NCI-H1299 xenografts. The tumor sections were stained with CD31 antibody (endothelial cells) and PDGFR-β antibody (pericyte), following with Alexa Fluor 488 Donkey anti-Rabbit IgG and Alexa Fluor 594 Donkey anti-Goat IgG staining. The DAPI was used to label nucleus. The images were photographed with a laser scanning confocal microscope, Scale bar: 20 μm.

We further validated the anti-tumor and anti-angiogenic effect of pristimerin using NCI-H1299 xenografts. Our results showed that pristimerin administration significantly suppressed tumor growth. The tumor volumes in the vehicle group grew from 102.75 ± 41.79 to 1206.34 ± 210.10 mm^3^, whereas the tumor volume in the pristimerin 0.2 mg/kg group grew from 97.12 ± 31.40 to 566.03 ± 148.19 mm^3^, the tumor volume in the pristimerin 0.4 mg/kg group grew from 109.82 ± 31.40 to 329.76 ± 118.72 mm^3^ (Fig. 5a). And the tumor weight in the vehicle group was 1.34 ± 0.28 g, which was much higher than that in pristimerin 0.2 mg/kg group (0.60 ± 0.087 g) and pristimerin 0.4 mg/kg group (0.28 ± 0.098 g) (Fig. 5b). And the body weight curve showed that pristimerin had minimal effect on the body weight of mice (Fig. 5c), suggesting that pristimerin has low toxicity towards tumor-bearing mice. And we further confirmed the anti-tumor effect of pristimerin using Ki67 staining, a cell proliferation marker. The result showed that the Ki67 index in pristimerin 0.2 mg/kg group and pristimerin 0.4 mg/kg group was obviously lower than that in vehicle group (Fig. 5d, e).

To understand whether the anti-tumor effect of pristimerin was attributed to its anti-angiogenic effect, we detected CD31 expression in the tumor tissue, a marker of MVD. The result showed that pristimerin treatment decreased CD31 expression, suggesting that pristimerin suppressed tumor growth of NCI-H1299 xenograft partially due to the anti-angiogenic effect. We also found that pristimerin administration inhibited the activation of Shh/Gli1 and its downstream signaling pathway, suggested by Gli1 and VEGFR2 expression decreased in pristimerin 0.2 mg/kg group and pristimerin 0.4 mg/kg group (Fig. 5d, e). Moreover, immunofluorescence staining showed that pristimerin administration reduced pericytes coverage in tumor tissues, indicated by that the PDGFR-β staining cells (a marker of pericytes) decreased around the tumor blood vessel (Fig. 5f). Altogether, our study demonstrated that pristimerin suppressed tumor growth and angiogenesis by inhibiting Shh/Gli1 signaling pathway in NCI-H1299 xenografts.

## Discussion

**Fig. 6:**
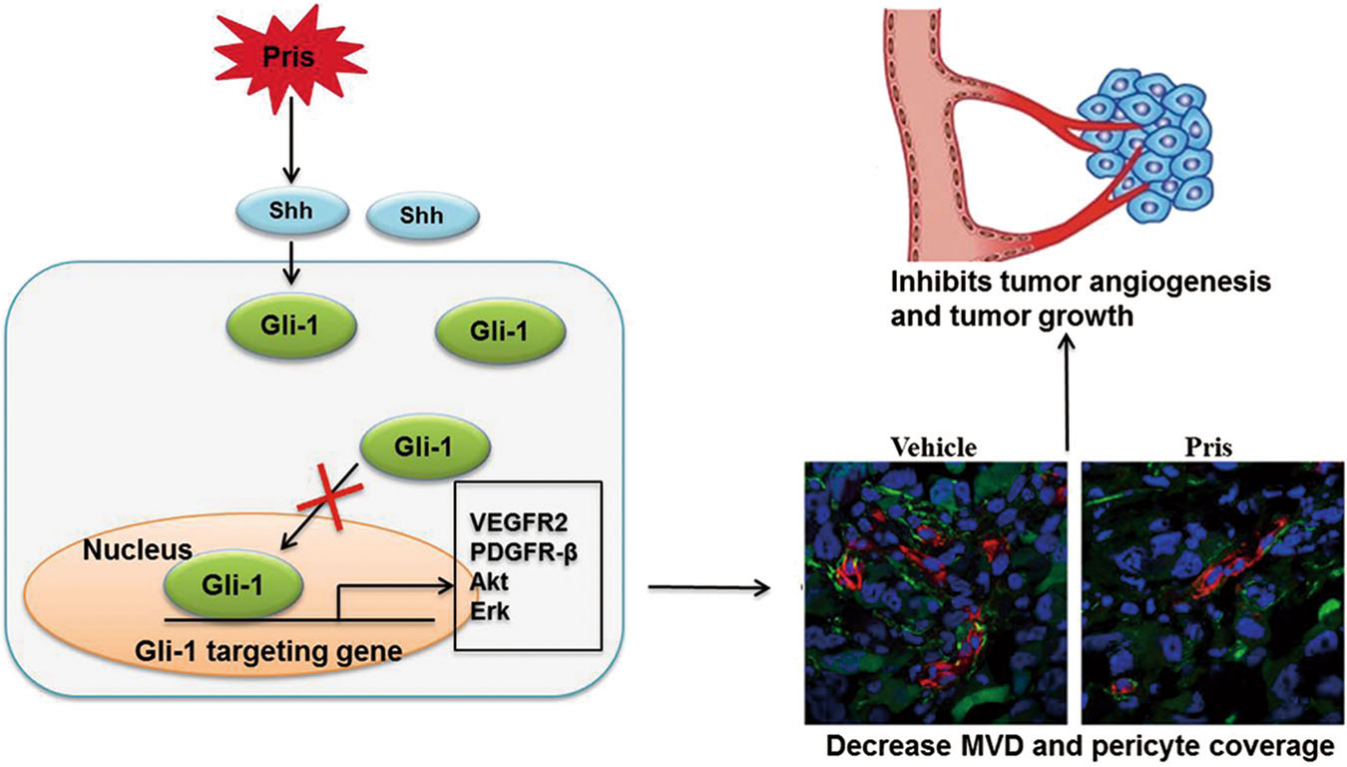
The proposed mechanisms of pristimerin-mediated anti-angiogenic and anti-tumor effects in NSCLC. Our data showed that pristimerin reduced Gli1 distribution in nucleus and then suppressed the activation of Shh/Gli1 signaling pathway, resulting in decreasing MVD and pericyte coverage.

Pristimerin has been widely investigated to validate its anti-tumor effects in many cancer models, but its effect on tumor angiogenesis and the underlying mechanism is not fully understood^30,34^. In the present study, we found that pristimerin suppressed Shh-mediated angiogenesis by restraining Shh/Gli1 signaling pathway and its downstream signaling pathway in vitro and in vivo. Furthermore, we showed that pristimerin inhibited not only Shh-induced endothelia cell motilities (proliferation, migration, invasion and tube formation) during angiogenesis but also Shh-mediated pericytes recruitment to newly formed endothelial tubes, which promotes the maturation of neovessels during the late-stage of angiogenesis (Fig. 6). Our study provides new insight into anti-angiogenic and anti-tumor mechanism of pristimerin. Our study also suggests that pristimerin is a novel candidate of anti-angiogenic agent for cancer therapy.

For decades, clinical efforts to develop angiogenesis inhibitors have largely focused on VEGF-mediated signaling pathway, and many of them have been applied in clinical practice. However, these angiogenesis inhibitors often lead to transient responses and side effects, including bleeding, proteinuria, hypertension, gastrointestinal perforation and drug resistance^35,36^. This may due to that tumor angiogenesis is a complex process regulated by many factors and they are able to compensate for each other when a single pathway are inhibited^6^. Thus, it is urgent to recognize novel drug target and develop novel inhibitors to combat tumor angiogenesis. Recently, several studies have showed Shh/Gli1 signaling pathway contributed to angiogenesis. Chen et al. demonstrated that Shh improved the MVD and promoted angiogenesis via enhancing VEGF expression after stroke in rats^18^. Yao showed that Shh involved in PDGF-BB-induced mural cells (MC) migration and recruitment into neovessels, resulting in the newly blood vessel maturation^19^. All these suggest that Shh/Gli1 is potential to be an anti-angiogenic target, but its role in tumor angiogenesis is still largely unknown. In this study, we showed that pristimerin suppressed tumor angiogenesis in NCI-H1299 xenografts via inhibiting Gli1 nucleus distribution in endothelial cells and pericytes, and then inactivating Shh/Gli1 and its downstream signaling pathway. In this regard, our study provides strong evidences for the vital role of Shh/Gli1 in tumor angiogenesis and indicates that targeting Shh/Gli1 is a potential strategy for suppressing tumor angiogenesis.

Pericytes is vital for the stabilization and maturation of neovessels during the late-stage of tumor angiogenesis by recruiting and attaching to newly formed blood vessel. Many of angiogenesis inhibitors targeting endothelial cells often result in drug resistance. Pericytes is identified as one of the most important factors that contribute to the drug resistance^19,37^. Several studies showed that Shh is critical for the maturation of neovessels through regulating migration and recruitment of MC^19,38^. In addition, Shh/Gli1 signaling pathway is vital for drug resistance in anti-tumor therapy and inhibition of Shh/Gli1 has been proved to remit drug resistance^39^. In the present study, we demonstrated that Shh stimulation promoted pericytes adhesion and recruitment to endothelial tubes and pristimerin suppressed tumor angiogenesis by inhibiting Shh/Gli1 signaling pathway. In this regard, our study provides strong evidence for the key role of Shh in pericytes. Our study also warrants further investigation into the potential of pristimerin for the treatment of anti-VEGF-resistant tumor. And we will evaluate whether pristimerin combats drug resistance of angiogenesis inhibitors targeting VEGF and explore the underlying mechanism in future study.

Pristimerin possess potent anti-tumor in many cancers, including breast cancer, prostate cancer, glioblastoma and hepatocellular carcinoma. The mechanism underlying the anti-tumor effect of pristimerin involves in cell cycle arrest, apoptosis, necrosis and autophagy that regulated by ROS/ASK1/JNK, HIF-1α, NF-κB signaling pathway^28,33,34,40^. And pristimerin has been shown to inhibit VEGF-induced tumor angiogenesis in breast cancer^30^. However, whether pristimerin suppresses tumor angiogenesis in other cancers and the underlying mechanism is still unclear. In this study, we showed that pristimerin exhibited potent anti-angiogenic in vitro and in vivo. Pristimerin suppressed not only Shh-mediated endothelia motilities in the early-stage of tumor angiogenesis but also pericytes recruitment to the newly blood vessel that is important for the maturation of blood vessel. Moreover, our further study revealed that Shh/Gli1 and its downstream signaling pathway contributed to pristimerin-mediated tumor angiogenesis. Our study provides new clues for further investigation of the anti-angiogenic and anti-tumor effects of pristimerin and contributes to understand the underlying mechanism of pristimerin-mediated anti-tumor effect.

In the study, we showed that Shh stimulation promoted the phosphorylation of VEGFR2 and pristimerin treatment suppressed Shh-induced the phosphorylation of VEGFR2 (Fig. 3d). And the translocation of Gli1 to nucleus was observed at 0.5 h whereas the phosphorylation of VEGFR2 was detected at 1 h, suggesting that Gli1 nucleus translocation was in advance of the phosphorylation of VEGFR2 (Supplementary Fig. 1). And considering the reports that Shh regulates the expression of many angiogenic growth factor, including VEGF, VEGFR2, Ang-1, Ang-2 and ENOS^22–24^, we speculate that pristrimerin may suppress the activation of Shh/Gli1 firstly and then its downstream VEGF/VEGR2 signaling pathway. And pristimerin-mediated anti-angiogenic effect may also partly depend on the activation of VEGF/VEGR2 signaling pathway as previous study described^27^. Further study is needed to explore how Shh/Gli1 regulates VEGF/VEGFR2 signaling pathway.

In conclusion, our study demonstrated that pristimerin obviously suppressed tumor angiogenesis and tumor growth in NCI-H1299 xenograft by regulating both the early- and late-stage of angiogenesis. Further investigation revealed that the anti-angiogenic effect of pristimerin was associated with the inactivation of Shh/Gli1 and its downstream signaling pathway. This study provides persuasive evidence for developing pristimerin into a novel antiangiogenic agent in cancer therapy.

## Supplementary information

Supplementary Figure Legends
