# Supplementary material for: Identification of a novel tumor angiogenesis inhibitor targeting Shh/Gli1 signaling pathway in Non-small cell lung cancer
Source: Cell Death Dis. 2020 Apr 14;11(4):232. doi: 10.1038/s41419-020-2425-0 (PMC7156472; doi:10.1038/s41419-020-2425-0)
Supplement: Supplementary file 1 — Supplementary Figure Legends [file 41419_2020_2425_MOESM1_ESM.docx]

**Supplementary Figure legends**

**Supplementary Fig. 1 Shh promotes Gli1 nucleus translocation and VEGFR2 phosphorylation.** After being stimulated with Shh for 0.5 h, 1.0 h and 2.0 h, the cells were collected for cytoplasmic extraction and nucleus extraction, and then for western blotting assay. Total protein was obtained using RIPA buffer.
